# Penpulimab and Anlotinib in PDL1 high-expression pulmonary giant cell carcinoma with cerebral metastases: case report and review

**DOI:** 10.3389/fonc.2024.1476205

**Published:** 2024-12-23

**Authors:** Minghong Xie, Yunlong Zhao, Xiaohua Hou, Ning Li, Sha Niu, Xinju Xu

**Affiliations:** ^1^ Department of Respiratory and Critical Care Medicine, First Affiliated Hospital of Henan Polytechnic University, Jiaozuo Second People’s Hospital, Jiaozuo, China; ^2^ Department of Pathology, First Affiliated Hospital of Henan Polytechnic University, Jiaozuo Second People’s Hospital, Jiaozuo, Henan, China

**Keywords:** pulmonary giant cell carcinoma, Penpulimab injection, Anlotinib, cerebral metastases, PD-L1 positivity

## Abstract

Pulmonary giant cell carcinoma (PGCC) is a rare subtype of non-small cell lung cancer (NSCLC) characterized by complex pathology, high rates of misdiagnosis or missed diagnosis, an aggressive clinical course, rapid progression, and poor prognosis. This case report describes a 67-year-old Chinese male with a left upper lobe lung mass, diagnosed *via* CT-guided lung biopsy as PGCC with symptomatic multiple cerebral metastases. The tumor showed strong PD-L1 positivity, and genetic testing revealed a TP53 exon 4 c.313G mutation. Treatment involved first-line therapy with Penpulimab injection combined with Anlotinib and concurrent cranial radiotherapy. Significant reduction in both the pulmonary and cerebral metastatic lesions was observed, with notable efficacy. As of June 2024, there has been no disease progression for 26 months, with the patient currently maintained on Anlotinib monotherapy. This case demonstrates the favorable efficacy of Penpulimab injection combined with Anlotinib in treating advanced PGCC. These findings indicate that this combination therapy may offer a promising new therapeutic option for this rare type of lung cancer.

## Introduction

PGCC presents clinically in a manner similar to other non-small cell lung cancers (NSCLC), yet it is distinguished by rapid proliferation and robust invasive behavior, leading to extensive multi-organ metastasis. The primary treatment for PGCC typically involves surgical intervention; however, due to the aggressive nature of the disease, surgery alone is often insufficient. PGCC exhibits high malignancy and poor prognosis, with most patients experiencing recurrence or death within 16-18 months post-surgery, and an average survival time approximating 12 months (1, 2).

Immunotherapy endeavors to reinvigorate antitumor immune cells and overcome tumor immune evasion mechanisms. Penpulimab, a novel IgG1 subtype monoclonal antibody targeting programmed cell death protein 1 (PD-1), has been exclusively approved in China. By binding to the PD-1 receptor, it blocks its interaction with programmed death-ligand 1(PD-L1) and PD-L2, thereby preventing immune cell phagocytosis and cytotoxicity through structural modifications in the Fc segment, and thwarting tumor immune evasion. Anlotinib is a multi-targeted tyrosine kinase inhibitor (TKI) known for its broad-spectrum inhibition of tumor angiogenesis and growth.

In recent years, combined immunotherapy and anti-angiogenic targeted strategies have shown promising outcomes. Previous studies have demonstrated that the combination of Anlotinib and Penpulimab as first-line treatment for hepatocellular carcinoma achieved an overall response rate (ORR) of 31%, a disease control rate (DCR) of nearly 83%, and a median progression-free survival (PFS) of 8.8 months, showing comparable efficacy to similar anti-angiogenic and immunotherapeutic combinations. Adverse events were manageable, with a satisfactory safety profile (3). In an Ib/II phase study of Penpulimab in 65 patients with advanced solid tumors, a median PFS of 12.6 months was reported, with an ORR of 12.3% (95% confidence interval [CI], 5.5%-22.8%). Complete response was achieved in 3 cases (4.6%) and partial response in 5 cases (7.7%) (4). A retrospective study evaluating the efficacy of Anlotinib combined with immunotherapy in 101 patients with advanced NSCLC found a partial response in 19 patients (18.8%), disease stabilization in 61 patients (60.4%), an ORR of 18.8% and a DCR of 79.2% (5).

Cerebral metastases remain a critical challenge in the management of NSCLC due to its high incidence and poor prognosis, with survival rates typically limited to just a few months. For patients with multiple cerebral metastases or those for whom stereotactic radiosurgery (SRS) is not feasible, radiotherapy is still considered the gold standard treatment (6). A retrospective analysis indicated that the median overall survival (OS) was 7.0 months (n=435) after stereotactic radiotherapy (SRT) and 3.0 months (n=1705) after whole-brain radiotherapy (WBRT). Additionally, 27% of SRT patients and 50% of WBRT patients died within 90 days of initiating radiotherapy (7). Reviews have suggested that immunotherapy and anti-angiogenic combination therapies might exhibit significant activity against cerebral metastases (8). Recently, in the era of immunotherapy, the use of PD-1 or PD-L1 inhibitors, either alone or in combination with chemotherapeutic agents, has demonstrated therapeutic activity against cerebral metastases in both NSCLC and SCLC patients (9, 10).

This case report presents a 67-year-old male with advanced PGCC and cerebral metastases who achieved remarkable clinical efficacy with a combined treatment of Penpulimab and Anlotinib, providing valuable insights for clinical management.

## Case presentation

**Figure 1:**
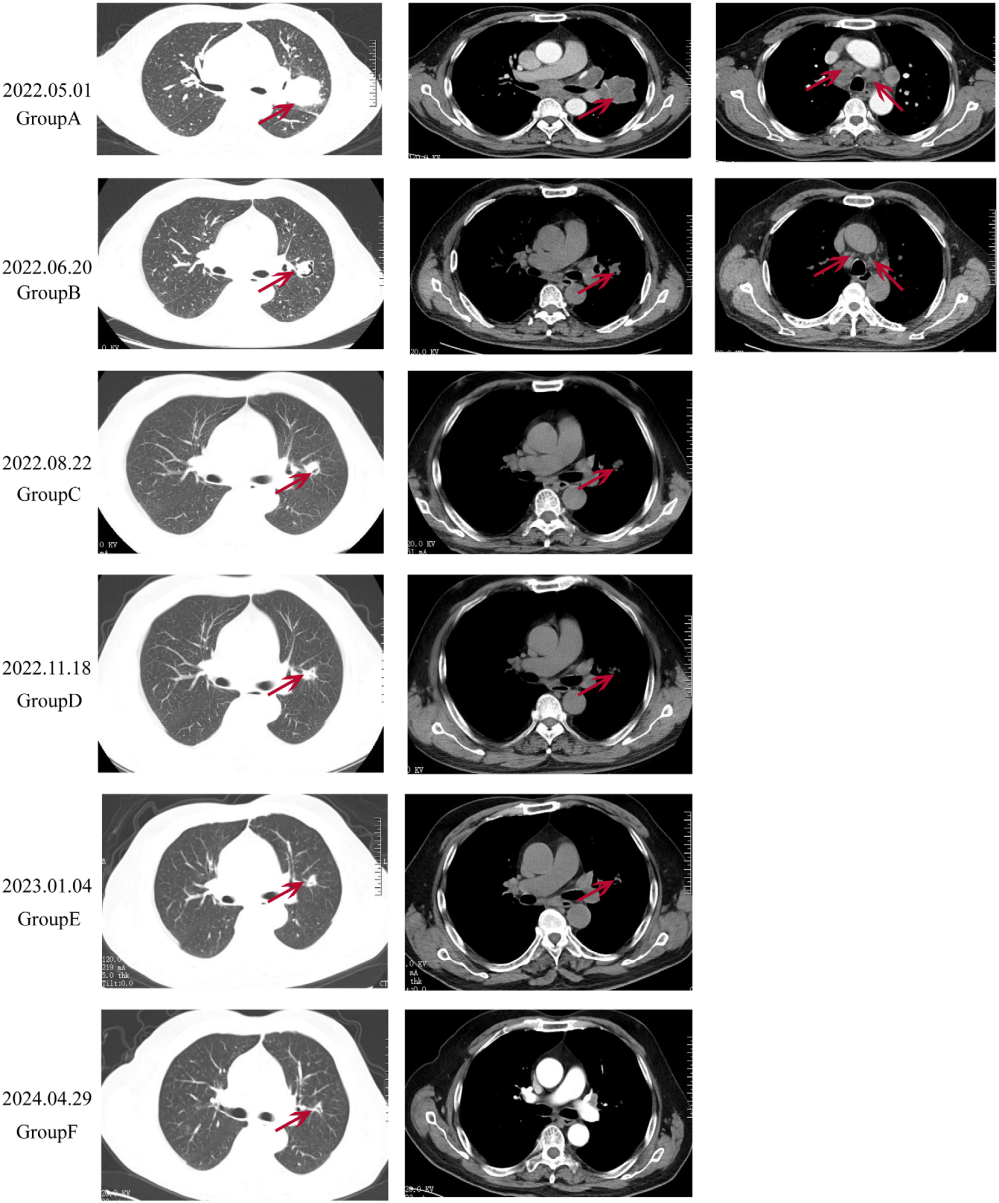
Radiological changes in the patient’s lung before and after treatment. **(A)**: Pre-treatment imaging showing a pulmonary mass measured approximately 49x50x55mm in size, with multiple enlarged and partially fused lymph nodes. **(B)**: After 2 cycles of treatment, the tumor size reduced to approximately 30x32x34 mm, and the mediastinal lymph nodes significantly decreased in size. **(C, D)**: Throughout the treatment, the lung tumor continued to shrink, and the mediastinal lymph nodes continues to decrease in size, with no mediastinal lymph nodes visible in **(E)**. **(F)**: During treatment, the tumor size was approximately 12x18x15 mm, with no significant enlargement of the mediastinal lymph nodes.

On April 22, 2022, a 67-year-old Chinese male presented with intermittent coughing for over 10 days underwent a chest X-ray at a local health clinic, revealing a lesion in the left lung. A subsequent chest computed tomography (CT) scan at a county-level hospital confirmed the presence of the lesion. The patient was then admitted to the First Affiliated Hospital of Henan Polytechnic University, where an enhanced chest CT scan showed an irregular soft tissue mass in the left lung with heterogeneous enhancement, measuring approximately 49mm x 50mm x 55mm. Multiple moderately enhanced nodular soft tissue density shadows were observed in the mediastinum, with some showing fusion (**Figure 1A**). The biomarker cytokeratin-19 fragment (CYFRA21-1) was elevated to 41.12 ng/ml (normal reference range <2.37 ng/ml). In adddition, the levels of biomarkers such as neuron-specific enolase (NSE) 、squamous cell carcinoma(SCC)、progastrin-releasing peptide(PROGRP)、carbohydrate antigen 242(CA24-4)、and carcinoembryonic antigen (CEA) were within the normal range throughout the disease and treatment course of the patients. The patient’s medical history included over 10 years of hypertension, a past case of hyperthyroidism which had been cured, and a 40-year smoking history of 20 cigarettes per day, with no family history of lung cancer.

**Figure 2:**
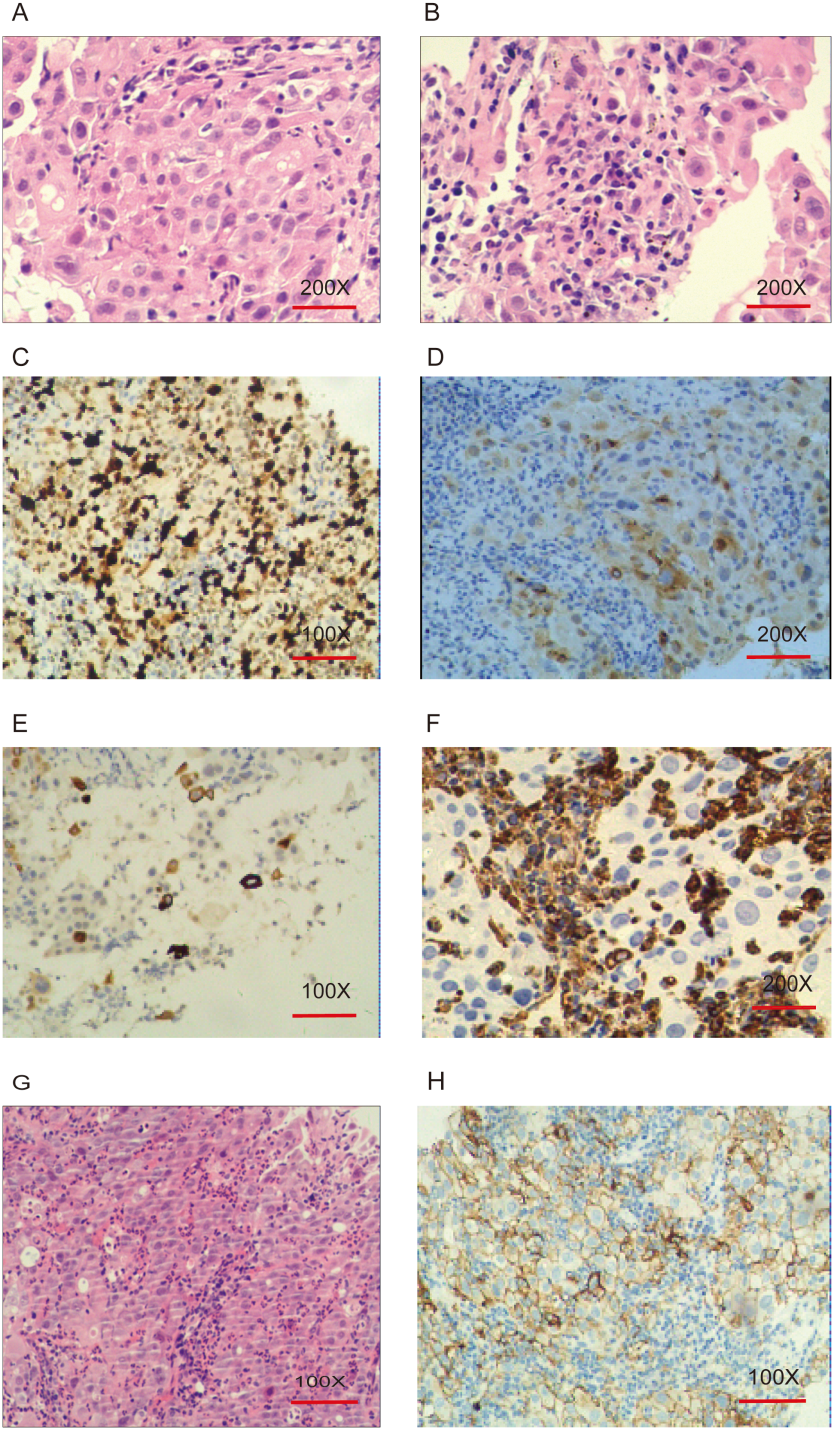
Pathological and immunohistochemical features of pulmonary giant cell carcinoma. **(A, B)**: Hematoxylin and eosin (H&E) staining, original magnification ×200. Tumor giant cells exhibit eosinophilic cytoplasm, poor cohesion, loose arrangement, large and irregular nuclei, prominent nucleoli, multinucleated giant cells, and neutrophilic infiltration. **(C)**: Ki-67 (70%+), original magnification ×100. **(D)**: CgA (+), original magnification ×200. **(E)**: CK5/6 (+), original magnification ×100. **(F)**: Vimentin (-), original magnification ×200. **(G)**: H&E staining, original magnification ×100. **(H)**: PD-L1 IHC, original magnification ×100. Tumor Proportion Score (TPS): 75% (TPS is defined as the percentage of at least 100 viable tumor cells showing partial or complete membrane staining for PD-L1).

Pathological examination of a CT-guided biopsy from the right upper lobe of the lung indicated NSCLC, immunohistochemically confirmed to be of lung origin. Microscopically, the tumor cells were characterized by eosinophilic cytoplasm, poor adhesion, loose arrangement, large and irregular nuclei, prominent nucleoli, multiple multinucleated giant cells, and infiltration neutrophils. Immunohistochemistry results showed high Ki-67 expression, and variable expression of Napsin A, TTF-1, P40, and CK5/6. In this case, focal weak positivity for CgA, focal positivity for CK5/6, and negative expression for Napsin A, TTF-1, P40, and Vimentin were observed, with high Ki-67 expression (70%) (**Figure 2**). These findings, combined with the morphological and immunohistochemical findings, supported the diagnosis of giant cell carcinoma.

**Figure 3:**
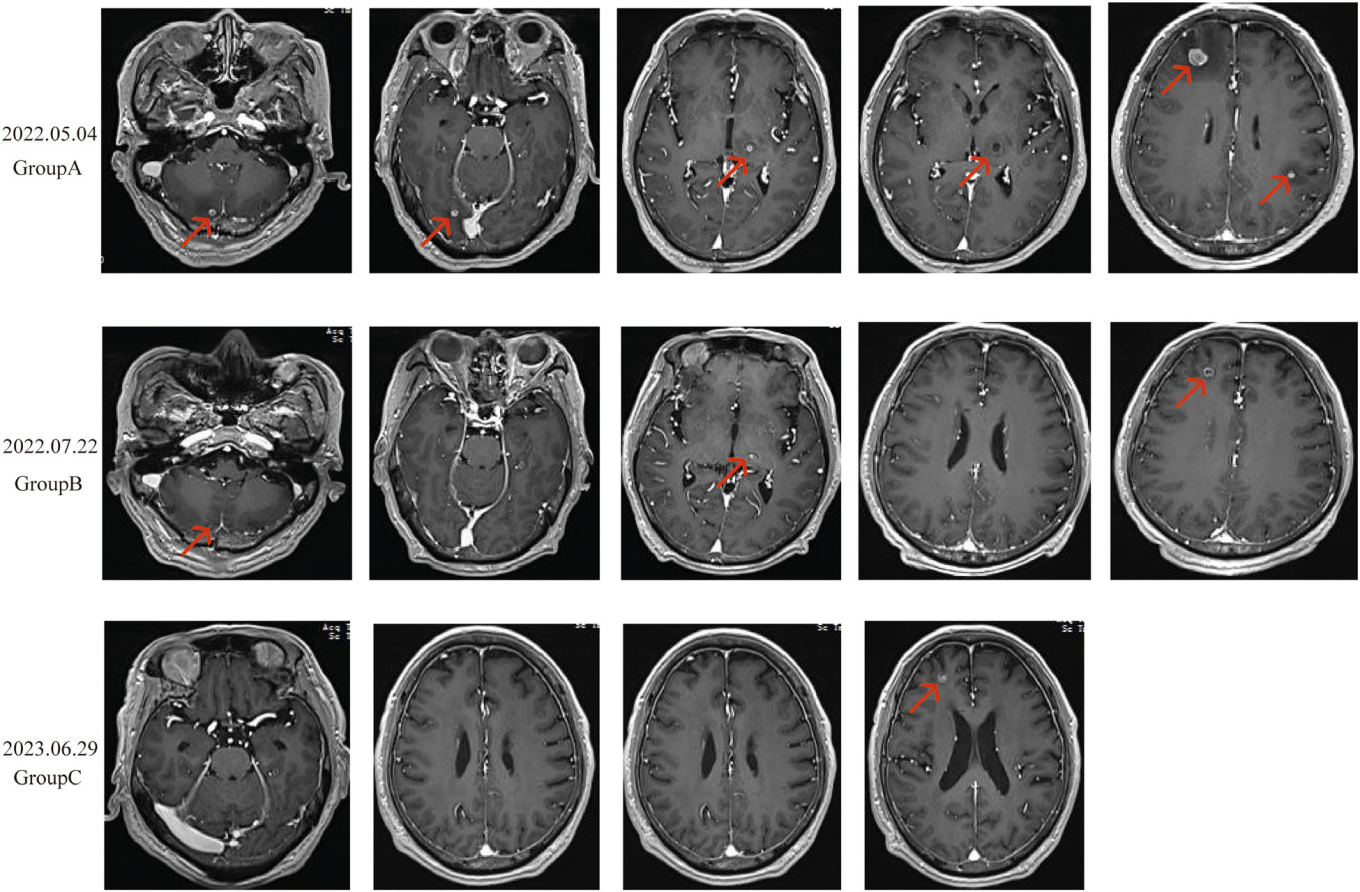
Radiological changes in brain metastases before and after treatment. **(A)**: Nodular and ring-enhanced abnormal signals in the right frontal-parietal lobe, left parietal lobe, left thalamus, and right cerebellar hemisphere. Multiple metastases are observed, with the largest lesion in the right frontal lobe, measuring approximately 11 mm in length, surrounded by small patches of edema. **(B)**: The brain metastases in the same locations showed significant shrinkage, with most lesions showing reduced enhancement. The largest lesion in the right frontal lobe measured approximately 7 mm in length. **(C)**: The metastases in the left parietal lobe, left thalamus, and right cerebellar hemisphere disappeared. The largest lesion, located in the right frontal lobe, measured approximately 7 mm in length.

Enhanced brain MRI indicated abnormal signals in the right frontal-parietal lobes, left parietal lobe, left thalamus, and right cerebellar hemisphere, showing nodular and ring-like enhancement with clear boundaries. The largest lesion was located in the right frontal lobe, measuring approximately 11mm in its longest diameter, with surrounding patchy edema, suggestive of multiple metastases (**Figure 3A**). Bone scans revealed no bone metastases, and enhanced abdominal CT scans showed no signs of metastasis. Based on the comprehensive evaluation of clinical and examination results, the patient was diagnosed with stage IV PGCC (T4N2M1a according to the TNM staging system).

Due to the patient’s symptoms of headache and nausea, priority was given to whole-brain radiotherapy (WBRT) at a dose of 40Gy/23f and a local lesion dose of 50Gy/23f, over 23 sessions. High-throughput sequencing of lung tissue specimens was performed to evaluate genes associated with lung cancer, revealing a TP53 exon 4 c.313G mutation. PD-L1 expression (TPS) was 75%. Based on the guidelines from NMPA, NCCN, and ASCO, and a review of public databases, the patient was subsequently given Anlotinib at 12mg daily, administered for 2 weeks and 1-week off, constituting a 3-week (21 days) cycle. This was combined with Penpulimab injection at 200mg, administered every 3 weeks (21 days), as a first-line treatment regimen. Excitingly, after two cycles of combined treatment, a follow-up chest CT scan showed a significant reduction in the size of the tumor in the left upper lobe of the lung (30x32x34mm) and the mediastinal lymph nodes (**Figure 1B**). A follow-up enhanced brain MRI indicated that the abnormal signals in the right frontal-parietal lobe, left parietal lobe, left thalamus, and right cerebellar hemisphere appeared as spot-like and ring-like enhancements with clear boundaries. The brain metastases had significantly reduced in size, with the largest lesion in the right frontal lobe now measuring approximately 7mm in its longest diameter (**Figure 3B**). The patient’s clinical symptoms of brain metastases were remarkadly relieved, and the quality of life improved significantly.

Two months later, a follow-up examination showed that the tumor had shrunk to 21x29x22mm, and the mediastinal lymph nodes had also significantly reduced in size (**Figure 1C**). Subsequent bi-monthly chest CT scans showed continued tumor shrinkage. By November 18, 2022, a follow-up chest CT scan showed that the tumor had significantly reduced to 12x19x17mm, with no significant enlargement of the mediastinal lymph nodes (**Figure 1D**). Follow-up enhanced brain MRI indicated that the metastases in the left parietal lobe, left thalamus, and right cerebellar hemisphere had disappeared, with the largest lesion in the right frontal lobe remaining at approximately 7mm (**Figure 3C**). According to the RECIST 1.1 criteria, the patient’s condition was classified as partial response (PR).

**Figure 4:**
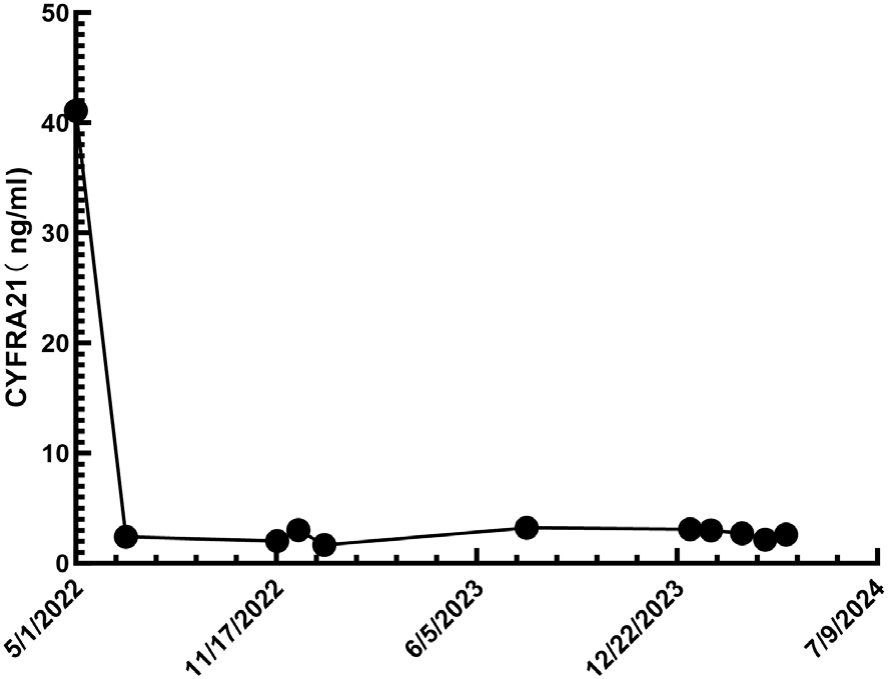
Changes in cytokeratin-19 fragment levels during treatment (normal reference range <2.37 ng/ml).

During follow-up chest CT scans. the tumor size remained stable, and on January 4, 2023, a chest CT scan showed the tumor remained at 12x19x17mm, with no significant enlargement of the mediastinal lymph nodes (**Figure 1E**). On April 29, 2024, a follow-up enhanced chest CT scan showed the tumor size was 12x18x15mm, with no significant enlargement of the mediastinal lymph nodes (**Figure 1F**). The CYFRA21-1 level had decreased to 2.61 ng/ml (normal range <2.37 ng/ml). According to RECIST 1.1 criteria, the patient’s condition was classified as stable disease (SD). The patient’s CYFRA21-1 level has stabilized between 2 and 3 ng/ml (**Figure 4**). Considering the sustained disease stability and the patient’s good tolerance, the Penpulimab treatment was lasted for two years and has now ended. The patient continues with maintenance therapy using Anlotinib monotherapy and is currently under regular monitoring and ongoing treatment.

## Discussion

According to the 2021 World Health Organization classification criteria, pulmonary sarcomatoid carcinoma (PSC) is a subset of NSCLC characterized by spindle or giant cell components. PSC can be classified into five subtypes: pleomorphic carcinoma, spindle cell carcinoma, carcinosarcoma, pulmonary blastoma, and pulmonary giant cell carcinoma (PGCC). These tumors, particularly those with spindle and giant cells, are highly aggressive and resistant to conventional treatments (11). PSC accounts for approximately 0.3%-0.4% of all pulmonary malignancies, with an incidence rate of three new cases per million annually. The disease predominantly affects male smokers, with a male-to-female ratio of about 5:1. Although PSC can occur at any age, the average onset age is between 50 and 60 years, which is younger than the average age for other NSCLC patients. PSC constitutes less than 1% of all lung cancers (12). Due to its rarity, data on treatment strategies and prognosis are significantly lacking (1), particularly for advanced PGCC, where randomized controlled trials and long-term follow-up data are scarce. This patient’s presentation with advanced disease and multiple metastases underscores the highly invasive and metastatic nature of PGCC.

Anlotinib is a multi-targeted anti-angiogenic agent that inhibits tumor growth by suppressing angiogenesis and inhibiting tumor cell proliferation and metastasis (13). The ALTER-0303 clinical trial demonstrated that Anlotinib is more effective than a placebo in the third-line treatment of advanced NSCLC patients. Compared to the placebo group, the Anlotinib group showed improved ORR and disease control rate (DCR) (ORR 9.18% vs 0.7%, P<0.0001; DCR 80.95% vs 37.06%, P<0.0001). Furthermore, Anlotinib significantly extended median PFS and OS compared to placebo (14).

Tumor immunotherapy, particularly immune checkpoint blockade, has achieved significant clinical success by inducing long-term regression in cases resistant to all other treatments. PD-1/PD-L1 inhibitors have shown promising therapeutic effects in various malignancies. PD-L1 expression is the only FDA-approved biomarker for guiding immunotherapy in lung adenocarcinoma patients. Results from the KEYNOTE-042 study indicated that first-line Pembrolizumab monotherapy continues to show long-term OS benefits and durable responses compared to chemotherapy, regardless of PD-L1 TPS, in PD-L1-positive locally advanced/metastatic NSCLC patients without EGFR/ALK alterations. The 5-year OS rate reached 22%, supporting the continued use of Pembrolizumab monotherapy as the standard treatment for previously untreated PD-L1-positive advanced/metastatic NSCLC (15). In patients with high PD-L1 expression (at least 50% of tumor cells) and advanced NSCLC, Pembrolizumab has demonstrated longer PFS, OS, and fewer adverse events compared to platinum-based chemotherapy (16). Retrospective studies and case reports have found that PD-1/PD-L1 inhibitors exhibit significant efficacy in PSC patients with high PD-L1 expression (17, 18). The high expression of PD-L1 provides a biological basis for the use of immunotherapy in PGCC patients.

Taking the tumor het erogeneity in human cancers into account, meticulous combined-modality treatment may be necessary to achieve breakthroughs in current cancer therapy for advanced refractory tumors.Clinical trials and studies have demonstrated that the synergistic antitumor activity of anti-angiogenesis combined with immune checkpoint blockade can enhance the treatment outcomes in various solid tumors (19–22). Anti-angiogenesis therapy can inhibit negative immune signals by increasing the ratio of antitumor/pro-tumor immune cells and reducing the expression of multiple immune checkpoints. Concurrently, immunotherapy can restore the immune-supportive microenvironment and promote vascular normalization (23). Therefore, the combination of anti-angiogenesis therapy and immunotherapy can synergize, improving therapeutic efficacy (24). The patient in this case achieved significant clinical benefits from the combination of immunotherapy and Anlotinib, consistent with previous study findings.

Brain metastases (BM) represent the most common form of intracranial malignancies in adults and are associated with poor prognosis (25). Lung cancer is the most frequent primary tumor leading to brain metastases (26). Increasing preclinical and clinical research indicates that combination therapies have significant antitumor activity; however, the efficacy against brain metastases remains unclear due to the stringent selection criteria in most clinical trials. In the era of immunotherapy, accumulating evidence supports using immune checkpoint inhibitors (ICIs) for treating NSCLC brain metastases in the absence of actionable driver mutations (27). In a multicohort study (NCT02039674) involving 25 patients, 4 (16%) had brain metastases, with an ORR of 56%, including 1 (4%) complete response and 13 (52%) partial responses. The median PFS was 7.1 months, and the median OS was 16.7 months (28). A retrospective analysis suggested that Pembrolizumab is effective in previously untreated NSCLC brain metastases patients with high tumor PD-L1 expression (29). The ARIO study assessed the outcomes of radiotherapy in NSCLC patients with brain metastases receiving ICIs (n=100) versus those not receiving ICIs (n=50). The study found that patients undergoing combined treatment had longer intracranial PFS (p=0.007) (30). Patients receiving radiotherapy (SRS/SRT) concurrently or after the initiation of ICIs achieved the greatest benefits, with most studies indicating that concurrent treatment improves OS (31).

In this study, a TP53 mutation was identified. TP53 mutations are the most common genetic alterations in PSC, with approximately three-quarters of patients harboring it. P53R2, a downstream target gene of TP53, comprises 9 exons and 1 intron and shares sequence homology with TP53. It plays a dual role in cancer regulation, including tumor suppression by promoting apoptosis and inhibiting cell proliferation; these pathways depend on the P21 signaling pathway. High levels of P53R2 protein are associated with poor OS. The presence of P53R2 is a significant factor associated with poor prognosis in PSC patients, and its expression is closely related to the occurrence, development, and progression of PSC (32).Generally, mutant TP53-carrying tumors reveal poorer sensitivity to conventional chemotherapy and have worse prognosis than wild-type tumors. This is a possible reason for chemotherapy resistance of PGCC (33).TP53 mutation-reversing drugs may be an effective option for the treatment of this disease in the future. However, currently targeted drugs for TP53 mutation have not been marketed. Although several drugs have shown significant efficacy in clinical trials, more trials are needed to verify. Basic studies have shown that TP53 mutations transmit signals conducive to tumor growth by promoting VEGF-mediated cell migration, angiogenesis, and metastasis or by overcoming ETS1 regulation (34). Therefore, VEGF pathway blockade may be effective in the treatment of this mutant tumor.

Tumor mutational burden (TMB) is also a useful biomarker for ICI response in advanced NSCLC (35). However, its value in brain metastases remains unclear. A retrospective study found that PSC patients with high TMB and TP53 mutations who received ICIs exhibited a longer survival trend compared to those with low TMB (18 months vs. 1.84 months) (23). This observation, however, has not yet been evaluated in advanced PGCC. The patient in this case has been treated for over two years, with follow-up enhanced brain MRI showing stable disease, suggesting that the combination of Penpulimab and Anlotinib may be effective against brain metastases. This indicates that the integration of immunotherapy, anti-angiogenic therapy, and cranial radiotherapy could provide additional benefits for patients. Further investigation is warranted to substantiate these findings.

In this study, the combination of Anlotinib, Penpulimab, and cranial radiotherapy was employed as a first-line treatment, resulting in long-term disease stability and significant efficacy. Therefore, Penpulimab combined with Anlotinib offers a promising new treatment option for untreated advanced PGCC or those with concurrent brain metastases, improving prognosis.

The patient, diagnosed with advanced PGCC, was confirmed *via* a small biopsy specimen from a lung puncture. Literature review indicates that small biopsy specimens are acceptable for diagnosing advanced NSCLC in clinical practice (36).

The primary adverse effect observed in this patient was Grade 1 hypothyroidism, which was attributed to Anlotinib. Despite this, thyroid function remained within normal reference ranges and was effectively managed with oral levothyroxine. No Grade 3-4 adverse events were observed. The optimal duration of maintenance therapy with Penpulimab remains undetermined, although clinical studies typically recommend a period of two years. Currently, the patient maintains a good quality of life, with continuous partial response (PR) and a survival period exceeding 26 months. Ongoing Follow-up will continue to monitor the patient’s condition.

To our knowledge, this is the first report demonstrating the efficacy of Penpulimab combined with Anlotinib in the treatment of advanced PGCC with brain metastases, high PD-L1 expression, and TP53 mutation. The combination of immunotherapy and anti-angiogenic agents presents a potentially promising strategy for treating PGCC. However, further studies are required to validate the efficacy and safety of this treatment approach.

## Data Availability

The original contributions presented in the study are included in the article/supplementary material. Further inquiries can be directed to the corresponding author.
